# Complexity in cancer biology: is systems biology the answer?

**DOI:** 10.1002/cam4.62

**Published:** 2013-02-17

**Authors:** Evangelia Koutsogiannouli, Athanasios G Papavassiliou, Nikolaos A Papanikolaou

**Affiliations:** 1 Laboratory of Biological Chemistry, Medical School, Aristotle University of Thessaloniki54124, Thessaloniki, Greece; 2 Department of Biological Chemistry, Medical School, University of Athens75M. Asias Street, 11527, Athens, Greece

**Keywords:** Cell cycle, complex, cyclin-dependent kinase, cyclin-dependent kinase inhibitors signaling, module, network, oncogene, oncoprotein, system, tumor suppressor gene, tumor suppressor protein

## Abstract

Complex phenotypes emerge from the interactions of thousands of macromolecules that are organized in multimolecular complexes and interacting functional modules. In turn, modules form functional networks in health and disease. Omics approaches collect data on changes for all genes and proteins and statistical analysis attempts to uncover the functional modules that perform the functions that characterize higher levels of biological organization. Systems biology attempts to transcend the study of individual genes/proteins and to integrate them into higher order information. Cancer cells exhibit defective genetic and epigenetic networks formed by altered complexes and network modules arising in different parts of tumor tissues that sustain autonomous cell behavior which ultimately lead tumor growth. We suggest that an understanding of tumor behavior must address not only molecular but also, and more importantly, tumor cell heterogeneity, by considering cancer tissue genetic and epigenetic networks, by characterizing changes in the types, composition, and interactions of complexes and networks in the different parts of tumor tissues, and by identifying critical hubs that connect them in time and space.

## Introduction

The decoding of the three billion nucleotides that comprise the human genome opened the possibility to isolate and characterize every encoded molecule under different conditions in health and disease 1–3. Ultimately, the hope is that this will allow us to devise preventive or therapeutic approaches for disease 4,5. Predicting higher functions from the structural and biochemical features of isolated single molecules is difficult 6. Proteins and other gene products are seldom found in isolation and are primarily organized in multimolecular complexes (see Box 1 for definitions of systems, complexes, networks, and modules) that execute unique and distinct functions that can be isolated and examined experimentally. Some can be transcription factors/cofactors interacting with chromatin during target gene transcription, others are cytoplasmic ensembles of kinases/substrates arranged linearly and branched to other pathways from the membrane to the nucleus and back, participating in interacting signaling networks that ultimately integrate information that is manifest as functional modules, yet others form extracellular structures that aid in cell–cell contact and tissue organization. Complex intracellular or intercellular behaviors, such as DNA replication, chromosome segregation, cell growth and division, motility, and migration, arise from the interactions of those functional modules 7,8 in different tissue parts that are interlinked via hubs (important molecules making multiple connections) in the form of networks 9. Biomolecular module networks consist of biomolecules (or nodes) connected by links (edges or interactions) and can be of different types depending on composition, signal integration, and time. For example, there are protein–protein interaction networks having physical interactions as nodes 10, gene interaction networks 11, gene expression networks 12 (although, it should be stressed that not all coexpressed genes are necessarily coregulated or reverse) 13,14, transcription factor/cofactor-DNA regulatory networks 15,16, signaling networks 17,18, and networks that describe how metabolism and growth are linked 19. Just as biological complexity at different levels, such as the ecosystem level, can be described by the complexity of interactions within subsystems under study and can globally be described by the connectivity L (see Box 2 and discussion in the next section) 20, tumor tissues also can be analyzed in terms of the different subsystems comprising the tumor, such as the different cell types, the modules, and the networks in each one that allow their viability as a tissue.

**Box 1. Defining systems biology**

“Systems biology defines and analyzes the interrelationships of all of the elements in a functioning system in order to understand how the system works”. In biology, systems approaches aim to:

1. Analyze the thousands of genes/proteins and other molecules comprising the system simultaneously, under different conditions (global analysis vs. local, i.e., one gene/protein),
2. Analyze several levels of complexity: molecules, complexes, modules, networks, cells, etc.,
3. Dissect networks: protein–protein interactions, signaling, metabolic and gene regulatory networks,
4. Computationally model/simulate processes, and
5. Determine temporal, environment, and genetic/epigenetic changes affect functions.

**Definitions**

1. *System*: Any collection of biological entities (genes, proteins, miRNAs, etc.) that are under study. Systems can contain molecules that can participate in different complexes/networks or modules.
2. *Complexes*: Groups of many proteins (and other biomolecules) whose interactions are cotemporal and cospatial. Complexes form molecular machines with distinct biochemical functions and their compositions and interactions can change genetically/epigenetically, thus determining network and module functions.
3. *Network*: Networks are systems of interacting biomolecules (collections of genes, gene products, etc.) with distinct functional outcomes. Networks can consist of single proteins or collections of complexes forming a grid. Interactions can be physical, functional, etc. Network organization is described in terms of links and nodes (see Box 2).
4. *Modules*: Collections of genes/proteins or interacting complexes that participate in determining specific cellular functions through network formation. They can arise from different networks or nodal proteins engaged in functional interactions.

A molecular network is imprinted as a *graph* and could be considered as an ensemble of *nodes* (representing biomolecules) and part of them are connected with *links*, *edges* (representing interactions and relations of the biomolecules). In each cell, there are different kinds of molecular networks such as protein–protein physical interaction networks, protein–protein genetic interaction networks, regulatory networks, expression networks, signal transduction pathways, and metabolic networks (better characterized than the rest). All these are cross-linked and combined together constitute the cellular network [108].

**Box 2 Description of networks**

Biological networks can have different forms, are connected by *edges* (usually molecular interactions, e.g., transcription factor–DNA, protein–protein, etc.) into *nodes* (proteins or genes), and are characterized by the degree and degree distribution defined below 9:

*Degree*: The connectivity *k* for each node: it can be *k*_in_ or *k*_out_ (see below).

*Degree distribution* P(*k*): The probability that a node (protein) has *k* links.

*Power law distribution*: P(*k*) ~ *k*^−γ^, where γ is the degree exponent. It describes the “scale-free” organization of biological networks, which refers to the fact that most nodes (either single proteins or multimolecular complexes) in a network make few connections and only a few nodes (clusters) make multiple connections. The former feature may be responsible for robustness 52 in biological networks 106,107, whereas the latter may render tumors vulnerable to drug targeting.

*Clusters*: Proteins (nodes) within a network with more interactions among themselves (cluster). Described by density of connections Q = 2*m*/(*n*[*n* – 1]) (see 22 for details). This description is identical to that of ecosystem networks, which is governed by the same equation (see below).
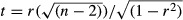

*Ecosystem complexity:* Defined by connectivity C(C): C = 2L/(N[N – 1]). It describes the actual food links divided by the number of all possible links. Notice that this equation is identical to equation Q = 2*m*/(*n*[*n* – 1]) proposed by Spirin and Mirny 22.

## Modular Organization of Tumor Tissue Complexity

Although no standard definition exists, a module can be defined as any subcellular unit (composed of complexes and their nested networks) having a distinct and unique task that remains robust, that is, remains constant and independent of perturbations or of individual biochemical parameters of any single molecule in the complexes that affect it (see Box 2). Modules consist of groups of biomolecules (genes, proteins, or gene products in general) that are found (often by different statistical approaches) to regulate as a unit a biological property or phenotype. These biomolecules can be hubs in networks and when they are linked together physically or functionally to perform a cellular function they then constitute a module.

The machineries that condense chromosomes in prophase and assemble them in metaphase, the DNA-repair or synthesizing enzymes, just to name a few, can be considered modules with distinct and separable functions. Modules also can be defined experimentally as groups of entities such as genes, proteins, or small RNAs that behave coherently, for example, during expression, and that contain gene products that affect similar or related functions. Additionally, there can be extended modules depending on how the components are organized 21. Other types of modules arise from interacting networks that are largely composed of complexes that also interact either simultaneously or in temporal sequence with multiple inputs/outputs manifested as complex functions, such as cell motility, division, etc. These are signaling modules and can have component complexes (or their important nodes) that interact both genetically and physically.

**Figure 1:**
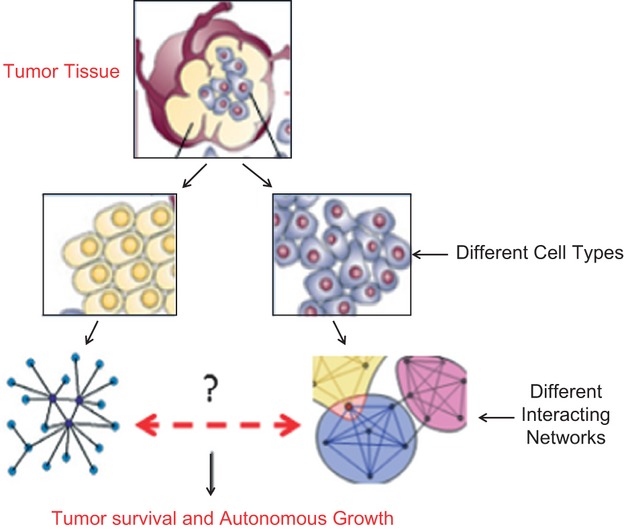
Representation of heterogeneous functional tumor tissue organization. Binary DNA information expressed as proteins/RNA is organized as intracellular networks (protein complexes, regulatory complexes, pathways, etc.) are identified with genomic/proteomic methods and deconvoluted into functional modules. Each cell type within tumors possesses different networks and modules that perform specific tasks, such as, for example, chromosome condensation, mitosis, motility, etc., and in a coordinated manner. Interactions between intracellular or intercellular modules via important hubs (protein nodes in different complexes or networks that interact functionally or physically with multiple other nodes in other complexes/modules) give rise to broad tumor interacting signaling networks. Many oncogenes are hubs and most are developmental genes. It is these tumor-wide interactions that endow tumors with robustness and survivability. The model suggests that identification of tumor-wide networks and the elucidation of intracellular and intercellular hub interactions allow redundancy in communication within the tumor mass and between tumor and adjacent “normal” tissues and could be prime pharmacological targets.

In tumor cells, modules like those previously described, are different (see last section for examples from oncogene literature) and largely control tumor survival and spread. Thus, complex behaviors such as invasion, which are controlled by several different types of modules, are evidently regulated differently when, for example, they are executed by lymphocytes or metastatic cells as in normal cells the process is terminated after some time, whereas in tumor tissues there is continued execution. We suggest that the modular organization of signaling networks are differently organized in the two cell types (Fig. 1). It remains largely speculative how different cells execute complex final functions (proliferation, invasion, etc.) using the same primary genome sequence. There are, as we shall see, tantalizing clues both in the molecular and the more recent genomics/proteomics literature, which suggest that oncogene/tumor suppressor proteins and their complexes (with largely uncharacterized partners) are critical hubs in signaling networks as they control multiple pathways (and presumably their interlinked networks and modules) that affect tumor properties.

Systems biology dissects complexes, functional modules, and their networks at several levels (see Box 1); it seeks to define the composition of complexes and to find out critical nodes, that is, molecule(s) with the most connections/interactions within and between modules, especially those that control functional interactions with other modules 22 uncover the laws that govern their physical behavior and relate them, in a predictable manner to overall cell biological behavior 1. In cancer cells, defining how nodal molecules such as oncogenes or tumor suppressors or their products connect and thus potentially regulate networks of gross cellular behaviors through their control of numerous intra- and intercellular signaling pathways is critical for understanding their signaling networks. Some oncogene/tumor suppressor products (Myc, p53, or cell cycle inhibitors and kinases) already fulfill the criteria of being important nodes; however, understanding how they influence different pathways is limited by lack of (a) how they participate in different complexes and networks and (b) how their levels/mutation status affects complex composition and function within and between different cell types in tumor tissues.

Reconstructing molecular mechanisms in cancer on the basis of modules, networks, and complexes is a formidable challenge both because of the enormous numbers of components in interacting networks but also because of inherent difficulties arising from methodological imperfections. For example, mRNA expression analysis with microarrays does not account for regulation of gene expression at the level of mRNA stability, processing, and protein post-translational modification levels 23. This is particularly apparent in microarray applications in cancer research due to complexities arising from molecular and pathophysiological classifiers that are different 24. Another issue is that data derived from global approaches can be interpreted in multiple ways depending on the experimental or theoretical model used, rendering relevance and applicability to mammalian cells somewhat questionable. An additional complication is due to the different meaning of what constitutes a module. Thus, for example, in the cancer microarray literature, a group of genes found to be regulated in common under a set of conditions is considered a module 25. A second common definition of a biological module is that of Hartwell et al. 21 who define it as a collection of many types of molecules (proteins, RNAs, etc.) having discrete functions that arise from their interactions.

**Figure 2:**
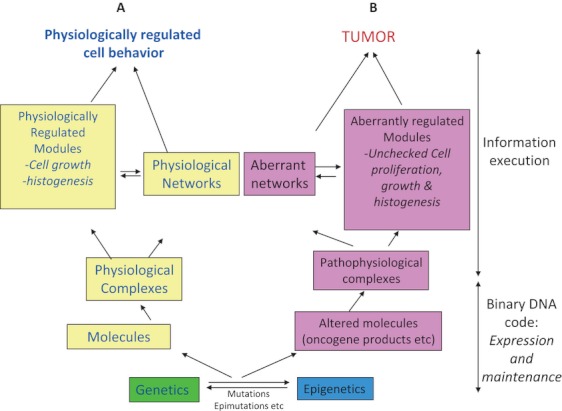
Simplified scheme for conceptual differences of different levels of biological organization in normal and tumor cells. (A) Recapitulation of different levels of organization and function in physiologically constrained cells. (B) In tumor cells, genetic (e.g., DNA mutations, chromosome translocations, etc.) and epigenetic alterations (aberrant methylation of CpG islands, acetylation of histones, etc.) generate altered biomolecules (activated oncogenic proteins, inactivated tumor suppressor proteins, or aberrantly regulated micro RNAs). Altered molecules form pathophysiological complexes that differ in quantitative and qualitative aspects from equivalent complexes in normal cells in composition due to over- or underexpression/inactivation of oncogene/tumor suppression genes, respectively, and are influenced by cell type and signals such as deregulated expression of other oncogenes/tumor suppressor genes, cell–cell interactions, etc. These complexes form “aberrant” signaling and other networks that regulate each other reciprocally and ultimately reorganize modules in novel ways that confer cells with autonomous growth and spread. Oncogene/tumor suppressor proteins may be important hubs in clusters that make multiple (functional and/or physical) connections with nodes in modules that, to a large extent, control tumor cell behavior. It is currently unclear how oncogene/tumor suppressor products and associated proteins in their complexes are organized into networks. However, experimental evidence suggests that they may in fact be organized as critical hubs in clusters between complexes, networks, and modules as (a) some are mutated in most cancers (e.g., Ras and Myc in ~30% and p53 in ~50% of all cancers), (b) their overexpression/mutation affects multiple pathways (and systems/modules) required for cell growth and proliferation, and (c) their effects depend on cell context. For example, their forced expression in normal cells causes growth arrest or apoptosis whereas in transformed cells they favor tumorigenesis, suggesting that their multiprotein complexes differ between normal and tumor cell populations.

We suggest a simple conceptual model which proposes that molecules, time- and/or signal-dependent complexes and networks that are formed within and between cells within tumors and between tumors and other organs, are organized hierarchically (nested networks) with feedback and feedforward interactions dictated by genetics and epigenetics (Fig. 1). The model suggests that in tumor cells, genetic/epigenetic-driven multimolecular complexes form altered networks 9 and modules 22 (as defined by Hartwell and colleagues). In turn, networks and modules determine how larger assemblies of functional modules (provisionally termed ultramodules, such as cell cycle progression, arrest or apoptosis, motility and invasion) become aberrantly operative in cancer cell behavior (Fig. 2). Changes in spatiotemporal complex composition are critical because they largely determine how molecules interact in altered modules and networks and how the function of complexes are changed 26. Finally, we provide support using examples from the cell cycle and Myc oncogene literature as well as from derived networks based on yeast and cancer cell genomic/proteomic data. The model aims to (a) provide a simple conceptual picture of functional organization and (b) make oncogene/tumor suppressor protein function (derived from experimental pathways data) more accessible to network modeling by reconciling protein pathway data with theoretical principles (e.g., node, edge, etc., vis-à-vis oncogene/tumor suppressor protein control of multiple pathways) and help in finding drug targetable nodes.

## Complexes, Signaling Networks, and Functional modules

The current revolution in our concepts and practices in analyzing complex biological functions has different conceptual and experimental roots. Nonliving, organized physical systems can exhibit emergent properties that cannot be attributed to single molecules but rather to intermolecular interactions within the system 27. Work in bacteria has led to the realization that several systems, such as the motility apparatus, are modular in organization as their properties cannot be attributed to single components and are characterized by robustness, that is, an ability to resist changes in their function 28. This concept has taken root in cancer biology and efforts are in progress to describe the reorganization of functional networks in cancer cells using mRNA, miRNA, and protein expression data converted to useful models and interpreted in terms of networks 29,30.

Recent advances in cancer cell genomic and proteomic methods greatly facilitate genomic and epigenomic analysis of functional cancer cell networks in terms of interacting molecules in complexes and in their constituent modules, especially in terms of the modular organization of oncogene expression and its correlation with specific cellular behavior 31,32. Genetic and epigenetic networks are altered in cancer cells and they are therefore likely to reflect permanent changes in the types, numbers, and interactions of complexes and modules at several levels, including the cell cycle 33 signal transduction pathways, such as the Ras-MAPK pathway 34 and chromatin remodeling in regulation of gene expression 35–37, giving rise to autonomous function of several modules associated with cell growth and invasion. While there is ample evidence in the molecular cancer literature that cancer cells have different modular and network design 38 of cellular behavior reflecting differences in the types and composition of complexes (Fig. 2), it is unknown (a) how this organization arises, (b) how the levels of identified gene protein products change in their respective complexes, and (c) factors which determine the nodes that (proteins or other molecules) are important in communications between altered complexes and modules. An important aspect of biological networks is that they can have several different forms depending on whether they consist of some (tens) or many (hundreds to thousands) of genes, proteins, or other biological entities studied en masse. For example, simple phages contain only a few genes and proteins whereas simple or higher eukaryotes such as yeast, or mice and humans contain hundreds to thousands. It was therefore necessary for high-throughput data collection and analysis techniques to be developed, starting from automated DNA sequencers all the way to network-generating software (Table 1). Data generation using cDNA or oligonucleotide microarrays 39,40 permits the simultaneous examination of all genes expressed at any given time under various conditions. For example, genome-wide expression analysis has been applied to the study of the yeast *Saccharomyces cerevisiae* cell cycle module 41. Protein–protein interaction maps have been generated for yeast using the two hybrid method 42 covering about 80% of all proteins and revealing important nodes, which when functionally inactivated by mutagenesis were lethal. Central to analysis of functional complexes, networks, and modules is the concept of “system,” which is any collection of molecules under study and is distinct from module or network. System molecules can participate in different networks or modules. In analogy to the definition of a module, there are at least two informal system definitions, the first due to experimentalists 43 and the second to theoreticians 44,45 (reviewed in 46). From a practical point of view although it is important not only to define the components of any biological system and model their interactions but also (a) to test if perturbing identified nodes affect the function under study and (b) if the links and function of any known biomolecules such as oncogene or tumor suppressor proteins can be reconciled with what is known about them experimentally.

**Table 1:** Selected techniques in systems biology

Subject	Technique
1. Genome-wide gene expression	Microarrays: oligonucleotide/cDNA chips microRNA expression
Genome-wide promoter analysis (Regulomics)
2. Expressed proteins (various conditions)	Antibody/cell extract chips (Proteomics/Reverse proteomics)
3. Network	
Direct protein–protein interactions	Yeast 2-hybrid
*In vivo/in vitro* pull down
Protein–DNA interactions	DNA footprinting
*In vivo* cross-linking/immunoprecipitation (ChIP)
Gel shifts
Genetic protein–protein interactions	
Gene regulatory interactions	Genome-wide promoter analysis (Regulomics)
4. Comparative genomics/proteomics	Various computational models and techniques
Protein functional genomics	DNA motif-finding programs
Reconstruction with Boolean or Bayesian network methods

Some techniques utilized in global identification of all molecules involved in a system under study and the construction of interacting networks that can explain and predict phenotypes.

One approach to test if theoretically determined protein interconnections in their networks and experimental data agree has been applied by 22. Their theoretical method is similar to that used by ecologists to quantitate ecological complexity in terms of food web network interactions (see Box 2) 20. These investigators sought to establish functionally important nodes within and between complexes using yeast protein–protein interaction data on two functional modules 47, the cell cycle and the MAP kinase module. They identified sets of yeast proteins (clusters) that have more connections among themselves than with other proteins in the protein–protein interaction networks under study. They defined the density of a cluster as Q = 2*m*/(*n*[n – 1]), where *n* is the number of proteins in the cluster and *m* the number of interactions. Significantly, genes and proteins (identified as clusters) found in the same complex or module had a consistent biological function, suggesting that biologically meaningful networks and modules arise from distinct protein complexes identified by genomics and proteomics approaches. This further suggests that analyzing the role of oncogene/tumor suppressor proteins in their complexes and the networks and modules they form in tumor cells may also be fruitful in identifying how oncoprotein-driven complexes and modules are organized in tumor cell models in terms of clusters or nodes. The theoretical results were further confirmed by tandem affinity purification and mass spectrometry even though data exist for only 29 experimental yeast complexes. An alternative approach, called multiple parallel signature sequencing (MPSS) 48 was applied by Hood's group in detecting definitive gene differences in early (androgen-sensitive) and late (castrate-resistant) prostate cancer 38 and used to map the networks that are different between the two states. Among significant genes/pathways regulated differentially, they identified the insulin signaling and the NFκ-B pathways interconnected to the c-Jun and IL-1 receptor pathways as well as at least 112 transcription factors that were differentially regulated in late versus early stage disease. Nevertheless, it remains to be seen how known oncogenes and tumor suppressor genes (and their protein products) fit into the identified disease and stage-specific patterns of signaling networks and whether they are clustered nodes with significant regulatory roles. Recently, Riera-Fernandez et al. 46,49 developed Markov–Shannon entropy models to evaluate connectivity (Lij) quality for network nodes in complex networks with an accuracy of nearly 76%. Notably, the models were predictive for different types of networks suggesting that it may be applied to mixed networks of biological interest.

According to network theory, a system can be described mathematically by its main components (nodes or hubs) and their connections (links). In addition to the above properties, many networks follow a power law distribution and are scale free, their clusters or nodes being quantified by the equations Q = 2*m*/(*n*[*n* – 1]) and P(*k*) ~ *k*^−γ^, which describe how networks are interconnected 9,50 (for definitions and a description, see Box 2). Thus, it would be informative to construct MYC or other oncogenic protein networks in model systems using data from real tumors and determine if the value of γ, and by extension of P(*k*) for oncogenic proteins such as MYC correlates with their experimentally uncovered influence on multiple cell pathways affecting proliferation and growth. Under this hypothesis, the lower the value of γ (within experimentally defined limits), the more important the protein would be predicted in networking and pathway control. In the case of protein complexes, the nodes are individual proteins and the links are their interactions, whereas for networks and their modules the nodes can be either individual proteins or groups of highly connected proteins both within and between modules that can determine functional outcome (see Fig. 4 for a schematic outline depicting experimentally determined connections of the Myc, p53, and TGFβ pathways). These pathways do not necessarily reveal the networks involved as they include only a limited number of nodes. In this respect, it is reasonable to suggest that oncogene products that are known to participate into different complexes and to affect several intracellular networks, such as MYC, RAS, or tumor suppressor proteins such as p53 and pRB, may qualify as important nodes with critical intracomplex and internodule links. Other potentially important nodes, making critical links may be inhibitors of the cell cycle, such as p21, which are also frequently mutated and inactivated in many cancers and for which we have some experimental information on how their levels may change the function of known cyclin-dependent kinase oncogenes (see last section for discussion). Moreover, other protein partners in the complexes and modules of these oncogene/tumor suppressor proteins also can be important in network dynamics and function; however, their role remains to be discovered. An example is the adaptor protein p40 in the ING1/Sin3A chromatin remodeling complex that controls growth and possibly metastasis in human cancer cell lines 37. Until now the complexes formed by these proteins, their composition and function in network and module formation during tumorigenesis remain incompletely characterized.

**Figure 3:**
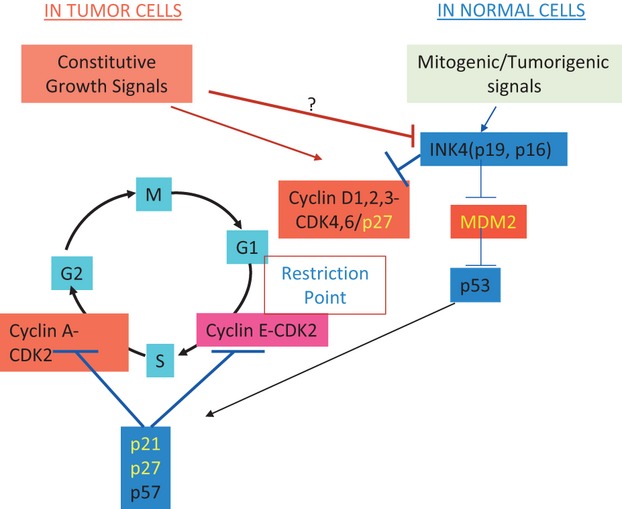
Schematic depiction of a eukaryotic cell cycle module with some critical regulators. Cyclin D1/Cdk4,6 complex formation and function depend, among other factors, on the levels of the Cdk inhibitor p27. Surprisingly, at relatively low levels, p27 aids in complex formation and stabilization (corroborated by the fact that p27 haploinsufficiency is found in tumors) whereas at higher levels – as in quiescent cells – it inhibits complex formation. It is unknown how complex formation and function is affected and how the signaling network that controls cell cycle progression is reorganized in terms of the participation of critical hub proteins in the depicted complexes. Thus, when INK genes are mutated, MDM2 and cyclin D1/Cdk4,6/p27 complex composition and function changes but it remains to be determined how their signaling networks are reorganized.

**Figure 4:**
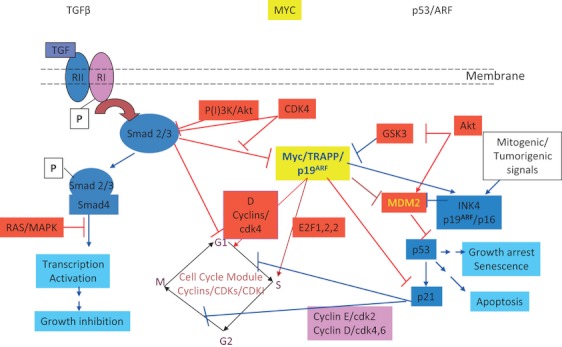
Schematic depiction of c-Myc functional interactions with other pathways (for full discussion, see section Induction–Reversion Tumorigenesis in Animal Models by Oncogene-Driven Cancer Network Formation). Blue arrows and sticks indicate (tumor) growth suppressive, functional interactions, and red tumorigenic interactions. Identifying Myc and other oncogene networks, especially in the context of others such as the p53 or Ras, in tumor samples using multidimentional analysis 98 promises to illuminate how oncogenes and tumor suppressor proteins are functionally networked in tumor cells and, in turn, how these networks determine cell growth autonomy. Key steps include determining which oncogenes or associated proteins in complexes and networks are important hubs or clusters and if so whether perturbing them is pharmaceutically feasible. Note: A pathway is different from a network in that it refers to an experimentally determined series of events that lead to a functional outcome as measured by commonly accepted methods. In contrast, a network is a grid of interactions between components of a system under study and can represent (a) direct structural interactions between proteins in a complex or a cluster, (b) functional gene–gene or genetic protein–protein interactions (obtained from DNA or protein microarray experiments). Genetic protein–protein interactions do not imply direct physical interactions between network-associated proteins.

The cell cycle, cell migration, cell division, and other cellular phenotypes can be considered as supramodules, consisting of smaller modules (e.g., chromosome segregation, kinase network signaling, DNA replication, etc.), whose fate is under the control of several networks consisting of protein and other complexes that are in turn regulated by small molecules (e.g., nutrient availability) and influenced by the relative levels and malfunction of activated oncogenes or tumor suppressor genes and proteins (see Figs. 3 and 4, a property that was also recognized in bacterial systems 52,28. In tumor cells, robustness is manifested in their redundant heterogeneity, the presence of diverse mutations in individual genes, in the ability to withstand and evade attack by the immune system and resist chemotherapeutic attack, and is among the most significant in contributing to growth and survival Fig. 4). Complexes and the networks they participate in are controlled by genetic, epigenetic, and spatiotemporal signals and are characterized by robustness. Another property of complexes is that they can share components, which can be members of different clusters. This property is reflected in the functional heterogeneity exhibited by oncogene proteins, such as MYC or RAS, to induce growth arrest in some cells and transformation in others. This property probably reflects their participation in several different complexes and is manifested in their ability to regulate many growth, survival, and invasion networks. Several transcription factor/cofactor complexes, which also include chromatin modification activities, share common adaptor proteins that act as scaffold or complex adaptor proteins 37. Several oncogene and tumor suppressor gene products such as MYC*,* MDM2, and pRB can participate in interactions with multiple partners and are members of different complexes and presumably can affect different modules, also in response to cell cycle stage, DNA damage, and other signals. Cyclin-dependent kinase (Cdk) inhibitors (CdkI) such as p27 also modify the functions of the cyclin/CDK complexes depending on their levels. Intriguingly, at low levels they seem to activate kinase activity whereas at higher levels they inhibit it 26,54. The levels of CDKI proteins can change due to mutations or to functional inactivation by overexpressed oncogenes.

## Complex Formation Driven by Differences in the Levels of Key Proteins and Implications for Functional Network Formation

The supramodules of cell cycle progression and cell division intrinsically are governed by the sequential activation/inactivation of multiple serine-threonine kinases called cyclin-dependent kinases (Cdks) in association with cyclins 55, as well as other regulatory proteins in complexes that control specific stages called restriction points 56 (Fig. 3). Cdk-cyclin/regulatory protein complexes in cell proliferation are critical because they have been found altered (mutated and/or aberrantly expressed) in virtually all human tumors. Although most of the canonical mammalian Cdk-cyclin complexes have turned out to be dispensable for cell proliferation due to functional redundancy 57,58, cyclins and CDKs are found to be altered in a majority of human cancers (e.g., cyclin D1 is overexpressed in 25% of human mammary tumors), suggesting that they participate in different networks that control mammary tumor cell proliferation and growth. Mammalian cell cycle progression is likely driven by networks and complexes that are cell type specific, and novel functions have been generated from studies using, for example, defective cyclin D1-mediated complex formation 59. Specifically, a mutant cyclin D1 having lysine instead of glutamic acid at position 112 has equal affinity toward its canonical Cdk4 and Cdk6 partners; however, although it is kinase defective, mice develop a normally expanded mammary epithelium (defective in cyclin D1 ablated mice and refractory to mammary tumorigenesis), suggesting that cyclin/Cdk4,6 complexes have additional functions or that they form additional complexes with uncharacterized functions. In support of this it is likely that cyclin D1/Cdk4,6 complexes are different in the two cases due to differences in p27 participation. It is currently unknown how cyclin D1/Cdk4,6/p27 complexes change in response to mutant cyclin D1 in target mammary cells. It is reasonable to suggest that it forms different Cdk4,6/p27 complexes whose network topology and function change as a result of fluctuating inhibitor levels. Some support is provided by analysis of trimeric cyclin D1/Cdk4,6/p27 complexes in cycling and quiescent cells. In quiescent cells, p27 levels are relatively elevated; however, as Cdk levels rise and their complexes accumulate, p27 is sequestered in their complexes 60. It turns out that relatively low levels of p27 (and p21) are required for formation of these complexes, and more surprisingly, p27 does not inhibit kinase function 54. Moreover, it is established in human breast tumors that low levels of p27 and elevated levels of cyclins D and E correlate with survival and have prognostic value, 61,62 conforming that p27 and cyclin E have crucial roles in the tumor-specific networks.

## Induction–Reversion of Tumorigenesis in Animal Models by Oncogene-Driven Cancer Network Formation

Determining how functional networks are reorganized in tumor cells using systems biology approaches is of paramount importance in developing network-based therapeutics. The involvement of identified networks must be experimentally confirmed using data from different sources aiming at defining how they render cells (a) immortalized, (b) defective in apoptosis, (c) able to metastasize 63, and (d) their circuitry delineated and critical nodes identified and tested for prevention and therapy 64. Proteomic approaches for defining oncogene/tumor suppressor protein complexes in tumors are still limited not only by technical issues but also, and perhaps more importantly, by tumor heterogeneity, implying that judicious choice of cellular or computational models will have to reflect the enormous variety of tumors sampled from patients. Effort will have to be invested in carefully identifying the signaling networks they form and the (supra)modules they regulate, such as the cell cycle, their interactions at the genetic and epigenetic levels, and ultimately the targeting of nodal proteins or protein complexes that comprise the modules in different types of tumors 65,66.

The realization that heterogeneity-derived, mechanistic complexity is the main problem to be solved in understanding and reversing tumorigenesis has been around for many years but only now are we able to dissect it in terms of experimentally compiled components (parts lists according to Ideker et al. 1) organized in multiple networks and leading to functional modules in tumor cells. Thus, it was observed early on that reversing oncogenesis in animal models required the introduction or removal of perhaps hundreds to thousands of genes by separation of specific chromosomes or by hybridization between normal and tumor cells 67–69, implying that different complexes interacting in networks are formed and reformed in these cells 70,71. These findings supported the notion that, in the long term, for chemical or genetic–epigenetic agents 72 to be therapeutically efficacious in patients, they should lead to global (genomic/epigenomic) reorganization of the multimolecular complexes and networks that control cancer cell behavior. Currently, cancer network (and presumably module deregulation/reregulation) formation and oncogenesis can be influenced in animal models by at least two approaches: first, with regulatable oncogene expression in animal models 73–75 and second, epigenetically by exploiting intrinsic signaling pathways, as, for example, in some leukemias, such as acute promyelocytic leukemia (APL), using the retinoid receptor ligand retinoic acid 76.

Work on oncogene proteins combined with gene chip microarray and proteomic analysis suggests that they possess properties that resemble those exhibited by nodes or clusters in theoretical networks reverse-worked from genomic and proteomic data analysis in yeast or, to a more limited degree, from human tumor models. Specifically, several of the well-known oncogene products Myc and Ras are known to (a) participate in many different multiprotein complexes, (b) control several different functional outcomes, such as proliferation, invasion, apoptosis, or growth arrest, and (c) induce different functional outcomes on cells depending on the cell type (normal vs. transformed, etc.), suggesting that this variety of functional effects might be the result of forming different complexes and therefore networks.

The c*-MYC* proto-oncogene, which is either amplified or overexpressed, is estimated to be involved in 20% of all human cancers and the c-Myc protein affects expression of nearly 15% of genes in genomes as disparate as flies and humans. The c-Myc transcription factor regulates gene expression by several transcriptional mechanisms, for example, by forming complexes with histone acetylases, DNA methyltrasferases, and basal transcription complexes in the regulatory DNA regions of target genes 77–80, and importantly, increased expression of the protein correlates well with cell transformation. The regulated genes belong to the cell cycle 81,82, metabolism 83,84, cell adhesion 85,86, and lately, to micro RNAs (miRNAs) 87, a class of small, genome-encoded RNAs (estimated to comprise approximately 2–4% of all human genes) with significant regulatory functions in normal and tumorigenesis-related gene expression. Like most proteins, c-MYC is never found in isolation within normal or tumor cells. It forms different complexes with a multitude of other regulatory factors whose composition can be cell cycle or mitogenic signal dependent. For example, c-MYC directly interacts with the general transcription factor TFIIB and assists in RNA polymerase III-dependent transcriptional activation of tRNA and 5S ribosomal genes 88. Also, it interacts with INI1/hSNF5, a protein member of the SWI2/SNF2 multiprotein complex that is involved in transcriptional regulation through chromatin remodeling activities and is required for c-MYC-mediated transcription activation 89. Although sequestration of c-MYC into different complexes and/or cellular compartments is probably one of many mechanisms to regulate formation of different c-MYC complexes, its ability to induce proliferation arrest or apoptosis in normal cells, or its synergism *in vitro* 90 and *in vivo* 91 in tumorigenesis in the context of Ras signaling could involve formation of novel complexes or rearrangement of existing ones, and Myc-driven networks that together lead to autonomous proliferation, growth, and invasion.

In mouse models, c-*MYC* overexpression causes tumor formation in different cell types and inactivation of expression reverses tumorigenesis. However, reactivation of c-*MYC* expression in the same animals reinitiates tumor formation in some organs such as breast 92 and pancreas, 93 whereas it induces terminal differentiation or apoptosis without tumor regrowth in others 94,95. Although the mechanisms are obscure, it is reasonable to suggest that, depending on tumor organ origin and the epigenetic profiles of the target cell types in the affected organs, the c-Myc protein forms different complexes, and therefore different networks, that reorganize modules that control cell proliferation, survival, and invasion differently in the different cell types and organs. Therefore, questions regarding the composition of c-Myc complexes and how they interconnect in network organization are critical in delineating how this transcription factor controls so many different tumor-associated phenotypes. Using data from different c-MYC-affected cell types it might be possible to generate expression, proteomic, and regulomic data that could illuminate the differences in the networks operating in different c-*MYC*-driven tumors in a manner similar to that used in validating the MCF10A-modeled wound response.

Ectopic c-MYC overexpression in normal fibroblasts can accelerate cell cycle entry but also induces apoptosis 96. This response depends on an intact ARF/MDM2/p53 tumor suppressor pathway, which monitors mitogenic and DNA damage signals impacting onto cells (Fig. 4) and whose module components are still largely unknown. In these cells, c-MYC activates/stabilizes ARF which subsequently inhibits MDM2 and stabilizes p53, leading to apoptosis 97. In contrast, in transformed cells, in which the ARF/p53 pathway is not functional, because of ARF mutations, apoptosis is evaded and tumorigenesis is favored. Key questions regarding how these proteins are networked in either case remains unanswered. For example, how does the presence of overexpressed MYC protein affect complex composition and function, especially in the context of other activated oncogene proteins such as RAS? How does progression of tumor formation abrogate TGFβ-mediated growth arrest and promote TGFβ-mediated metastatic behavior? How is SMAD2/3-mediated inhibition of MYC activity in its complexes altered in tumors (see Fig. 4)? It is reasonable to suggest that these functional outcomes are caused by reorganization of interacting MYC, p53, TGFβRII/SMAD2,3, and ARF complexes, which in turn generate novel networks and ultimately deregulate functional modules. Therefore, assessing MYC, p53, TGFβRII, and ARF networking by determining how they are interconnected could potentially lead to novel findings, including identifying promising targets (nodes) within their networks. Ultimately although, for useful target identification within c-MYC-regulated networks in human tumors and eventual clinical utility, it might be necessary to combine data from many different sources, including patient survival outcomes, histology, gene microarray, proteomic, and network data. This analysis requires the use of data from several different sources (higher data dimensionality 98), for example, in a manner similar to that used in validating the model for wound response in the human breast-derived, nontumorigenic MCF10A cell line 99.

## Large-Scale Integrated Genomic Analyses and Cancer Network Heterogeneity

Large-scale integrated genomic studies by the Cancer Genome Atlas Research Network have used mRNA, microRNA, promoter methylation, DNA copy number variations, and DNA sequencing of exons of coding genes in various tumors such as ovarian and breast cancer samples. Integrated genomic analyses have shed light on the landscape of molecular alterations that characterize ovarian cancer genomes and (not surprisingly) revealed that the p53 gene is mutated in nearly all samples and that there are at least four transcriptional ovarian subtypes 100. These subtypes also have three different miRNA subtypes and four promoter methylation subtypes. This suggests that the subtypes differ in the networks and subnetworks formed by the products of expressed genes (proteins, miRNAs, etc.). Although the number of mutations and altered genes is statistically rather low, the fact that tumor samples have different transcriptional signatures indicates that tumor cells and tissues are extremely heterogeneous, hence robust to general therapeutic approaches. Another important point that stems from this comprehensive study is that whereas the RB1 and RI3K/RAS pathways are functionally altered in 67% and 45% of samples, respectively, the existence of different transcriptional subtypes, three miRNA subtypes, and four epigenetic subtypes strongly suggests that the products of these genes, along with their functional partners, must differ in different subtypes. It remains to be found how their networks differ and whether their network properties are consistent with their predictive role. Analysis of breast cancer samples by the same approaches has revealed a similar picture, strengthening the argument that molecular heterogeneity is reflected in gene product and clinical heterogeneity 101. Interestingly, here too more than 10% of mutations in three genes (p53, PI3K, and GATA3) are found in all subtypes. An important next step would be to reconstruct p53, PI3K, or GATA3-centric networks in the different subtypes and dissect their links to other genes/gene products, particularly to hubs in order to identify novel connections between altered pathways.

Complexity in tumor cell organization is reflected in molecular heterogeneity as different networks (arising from mutations that are different in different regions of a tumor) are formed by a narrow range of the same mutated genes in different cell types within tumors and will be a challenge to personalized medicine and particularly to biomarker development. Work by Gerlinger et al. 102 has demonstrated that during tumor growth nearly two thirds of somatic mutations are absent or nondetectable in every tumor region. Notably, several tumor suppressor genes have undergone different mutational events in different tumor regions and also different tumor regions harbored different good and bad prognostic expression signatures. Therefore, therapeutic and biomarker development challenges will be formidable. One approach to address this state of affairs will be to identify the wiring of hubs in different tumor cells and regions and to devise hub-targeting methods aimed at preventing heterogeneity from evolving in a Darwinian manner. Hubs and bottleneck nodes in networks tend to be important for the functioning of cells and their targeting could prove valuable. Scaled network disruption has been recently proposed by Camacho and Pienta 103 as an approach emulating ecological species organization which takes advantage of the nested organization of such networks. In ecological networks, disruption of a single species (node) does not disrupt network organization and the same can be said of tumors which can be viewed as nested communities of different networks. This explains why single agents rarely lead to curative results. In contrast, where heterogeneity is limited, as in some leukemias (e.g., APL), single-agent administration often leads to better treatment outcomes if not curative results.

From a computational/bioinformatic point of view, the heterogeneity issue has been addressed by building databases which incorporate several different types of data that refer to a common property in tumors under study and which create connectivity maps between disparate data. ONCOMINE is one such database 104,105. It is worthwhile noting here that given that there has been a decline in funds available for cancer research and given the fact that cancer incidence is on the rise, the development and application of personalized treatments and the development of accurate biomarkers will be a difficult task ahead for basic scientists and clinicians.

## Conclusions

We have proposed a simple conceptual model to explain the hierarchical organization and possible functional interactions between multimolecular complexes formed by genetically/epigenetically encoded and modified molecules, the networks and modules they potentially form and how changes in their properties and composition (due to changes in the levels or mutation status of key molecules exemplified by p27) could explain tumor formation. The evidence for this model derives from several experimental and computational/theoretical sources. First, changes in the levels of cell cycle inhibitors in cyclin/CDK complexes control assembly and function of the respective complexes by determining progression or inhibition of progression depending on the levels of CDKI 54. Second, alterations in the levels of some oncogenic proteins (MYC or RAS) can have opposite effects on growth and proliferation depending on whether their levels increase in normal (growth inhibition, arrest, or apoptosis) or transformed cells (enhancement of tumorigenesis) 96. Third, based on combined theoretical and genomic/proteomic data in yeast models, proteins/genes that belong to the same complex or module exhibit correlated biological functions 22. Finally, regulated MYC overexpression/inactivation in mouse models suggests that, depending on the presence of different signaling networks/modules, oncoproteins that may be hubs form different complexes and consequently networks that affect cell behavior differently 75.

In addition to technical imperfections that generate systematic experimental errors, several issues will have to be clarified before systems biological approaches become fruitful in cancer research and find use in clinical applications. Central to this will be efforts aimed at identifying hubs between and within networks of oncogenic proteins that drive specific cancers, correlating these to actual tumor data and identifying intracomplex or network/module targets that control complex/network functional interactions using multiple sources of data. Clearly the identification of combined mRNA, protein, and miRNA signatures for tumor entities such as, for example, for invasion or proliferation would facilitate the discovery of novel connections and biomarkers. Analysis of these data and their reduction to as few parameters as possible capable of describing disease states will require progress in (a) the characterization of the role of known (and unknown) oncogene/suppressor proteins (and other hub proteins) in their respective tumor-specific complexes, which to date remain poorly defined, (b) the delineation of their networks in terms of functional tumor protein clusters, and (c) placing these proteins and their physical/functional partners in realistic clusters within and between networks, and (d) delineating how they interact in controlling the modules of cell proliferation, growth, and invasion.
